# Abortion law in the Crown Dependencies: liberalisation in the UK’s small, independent neighbours

**DOI:** 10.1093/medlaw/fwag030

**Published:** 2026-09-29

**Authors:** Jordan A Parsons, Alexandra Mullock

**Affiliations:** School of Law, Royal Holloway University of London, London, TW20 0EX, United Kingdom; School of Law, University of Manchester, Manchester, M13 9PL, United Kingdom

**Keywords:** Abortion, Crown Dependencies, Decriminalisation, Policy transfer, Telemedicine, Termination of pregnancy

## Abstract

There has been significant exploration of UK abortion law variation in recent years. However, this comparative research has not extended to the neighbouring Crown Dependencies. The Isle of Man, Guernsey, and Jersey operate in unusual constitutional contexts, with more autonomy than the UK’s devolved nations. Whilst they often match UK policy in many areas, this is not automatic. In this article, we examine recent progress the Crown Dependencies have made on abortion regulation. Following a brief constitutional background and overview of UK abortion law, we examine the differing ways all three Crown Dependencies have approached the question of abortion. Finally, drawing on these developments, we suggest a broader comparative approach in health law in the British Islands would be beneficial because of the significant social, legal, and medical links between the UK and the Crown Dependencies. We argue any characterisation of the Isle of Man, Guernsey, and/or Jersey as following the UK for policy direction is inaccurate where health policy questions involving fundamental moral choices are concerned. Instead, the Crown Dependencies have flexed their independence to show a pioneering approach to an important and morally sensitive matter of health law, providing useful insights other jurisdictions may benefit from.

## I. Introduction

There has been significant exploration of abortion law variation across the UK in recent years—and, indeed, the impact of devolution on myriad health matters. Whether in relation to the delivery of abortion care by telemedicine or partial decriminalisation in Northern Ireland, differing approaches to this traditionally controversial policy matter have been a point of interest to legal scholars. However, this comparative research has seldom been extended to consider the neighbouring Crown Dependencies. Whilst the Crown Dependencies are not part of the UK, they have close and important social, cultural, regulatory, and employment links with the UK, and so we suggest there is good reason to consider them in comparative work. On matters of healthcare, the links between the Crown Dependencies and the UK are particularly important because people living on the islands depend on the UK for access to many specialist medical treatments, including abortion. Examining the distinct approaches in each jurisdiction reveals how the Crown Dependencies have diverged and converged—from the UK and each other—whilst transitioning from very restrictive abortion laws to far more progressive approaches. We explore how these small jurisdictions have innovated whilst also being influenced by the UK on abortion policy. Our analysis suggests that the evolution of, and recent developments in, abortion law have been subject to a “push and pull” effect between the UK and Crown Dependencies.

The Crown Dependencies—the Isle of Man, (the Bailiwick of) Guernsey, and (the Bailiwick of) Jersey^1^—operate in unusual constitutional contexts. They enjoy far more autonomy than the UK’s devolved nations but remain subject to elements of oversight from the British Crown (rather than the UK Government). Whilst the Crown Dependencies often choose to match UK policy in many areas, this is *not* automatic and is subject to their respective legislatures opting to do so—whether by taking up permissive extent clauses or passing comparable legislation.

Now is a particularly opportune time to begin examining approaches to health law in the Crown Dependencies. Recent years have seen headline-grabbing progress on controversial matters such as abortion (as we will explore in this article) and assisted dying.^2^ Also, alignment across the UK and Crown Dependencies has been observed in organ donation.^3^ There has been much regulatory work to iron out practicalities of healthcare provision with the UK.^4^ Further, both the Isle of Man and Jersey have sought to overhaul their health systems after somewhat damning external reviews.^5^

The reasons why including the Crown Dependencies can enhance comparative health law that is currently focused on the UK largely centre on similarities and important links between the jurisdictions. Indeed, there is a recognised legal grouping of the UK and Crown Dependencies as ‘British Islands’, as distinct from the British Isles. In demographic and social terms, the populations of the UK and the Crown Dependencies have many commonalities. This comes partly from an historic and ongoing exchange of residents.^6^ Particularly in the financial sector, which the Crown Dependencies are renowned for, professionals are often recruited from the UK. Many senior civil servants and even elected officials were also born and raised in the UK. Whilst this is true of all the Crown Dependencies, it is especially true of the Isle of Man—perhaps in part owing to geographical proximity.^7^ For example, both the Chief Officer of the Department of Health and Social Care^8^ and Director of Public Health and Chief Medical Officer^9^ on the Isle of Man were both recruited from the UK.

As well as many of those working in law and policy in the Crown Dependencies coming from the UK, a shared history has resulted in close legislative alignment on many matters. Throughout this article, we explore aspects of the constitutional relationships between the Crown Dependencies and the UK which serve to highlight this. Certainly, the way abortion law has been framed and reconsidered has many similarities across the British Islands. The effect of this is that, inevitably, where reforms have been considered in the Crown Dependencies, many of the questions being asked are of equal relevance to the UK. The Crown Dependencies routinely consult with legal, policy, and healthcare expertise from the UK in seeking to answer such questions. This is essential because, in addition to Crown Dependency residents accessing treatment in the UK, healthcare professionals working in the UK and the Crown Dependencies share regulators (e.g., the General Medical Council and the Nursing and Midwifery Council). Consequently, when healthcare professionals are working across jurisdictions, or treating patients from another jurisdiction, they must abide by regulatory norms whilst also adhering to the different laws in each jurisdiction.

Whilst all these matters raise interesting points for comparison more generally, our focus in this article is on the changes to abortion regulation across the three jurisdictions. We begin with a brief constitutional background of the Crown Dependencies to provide important context to the lawmaking process. Then, after detailing abortion law across the UK as a point for comparison, we examine the differing ways in which all three Crown Dependencies have evolved their own. Finally, drawing on these developments, we suggest that adopting a broader comparative approach in UK(-adjacent) health law would be beneficial in order to assess how the distinct approaches across jurisdictions with similar social, cultural, legal, and health contexts impact their respective populations and healthcare professionals who may be subject to multiple legal regimes. As this article demonstrates, whilst any characterisation of the Isle of Man, Guernsey, and/or Jersey as following the UK for policy direction was once largely accurate, it is no longer the case on matters of health policy involving fundamental moral choices. Instead, the Crown Dependencies have flexed their independence to show a pioneering approach to some of the most important and morally sensitive health questions. We see this most notably in the Isle of Man where they have gone further and faster in progressing more liberal abortion regulation.^10^ Other jurisdictions—predominantly the UK and its composite nations—would benefit from considering the approaches taken in the Crown Dependencies, particularly amidst current developments over (partial) decriminalisation in England and Wales with the Crime and Policing Act 2026.^11^

## II. Constitutional background

We recognise that there is not widespread understanding of how the Crown Dependencies operate politically, nor the precise nature of their relationships with the UK. Evidence suggests that even politicians working within the Crown Dependencies may not fully appreciate the constitutional arrangements and obligations between the UK and their own jurisdiction.^12^ Here we provide a concise overview of the constitutional arrangements affecting lawmaking on each of the three islands.

The Crown Dependencies are not formally part of the UK but are recognised as British Islands (by the UK, at least).^13^ The other Channel Islands—Alderney and Sark—are part of the *Bailiwick* of Guernsey,^14^ as distinct from the *island* of Guernsey (our focus in this article). These islands are not, and have never been, British colonies nor British Overseas Territories.^15^ As *Crown* Dependencies, their relationship with the UK is uniquely vested through the Crown and thus the Sovereign. Their relationship is not formally with the UK Government, even if for practical purposes there is significant interaction with Whitehall. This unique constitutional arrangement was examined in *R (Barclay) v Secretary of State for Justice (No 2)*,^16^ when the Supreme Court confirmed that whilst the constitutional convention is to consult and legislate by consent, the UK Parliament can legislate for the Channel Islands (and by inference also for the Isle of Man^17^) if necessary. For example, to ensure their law complies with the UK’s international obligations. In practice, the legislative choices of the Crown Dependencies have not resulted in a pattern of UK interference.^18^ Morris has explored how the Crown Dependencies occupy a unique and highly favourable position, with ‘sweeping powers of self-government buttressed by a strong and universally observed constitutional convention that Westminster may only legislate for them after consulting with and obtaining the prior consent of the insular authorities’.^19^

People born in the Crown Dependencies enjoy British citizenship under the British Nationality Act 1981,^20^ and (along with the Republic of Ireland) all fall within the Common Travel Area, meaning no immigration control between the islands and the UK.^21^ This is a crucial factor when considering health policy because of the importance to Crown Dependency residents of accessing specialist treatment in the UK, including later-term abortion.

The UK’s responsibility towards the Crown Dependencies primarily concerns defence and international representation.^22^ Whilst each Crown Dependency has its own legislature, all legislation requires Royal Assent by the UK Crown. The UK Ministry of Justice^23^ has oversight of the UK’s relationship with each of the Crown Dependencies,^24^ including examining all legislation to ensure that there is no conflict with ‘fundamental constitutional principles’ or with any international treaties,^25^ such as the European Convention on Human Rights. So, whilst abortion is an issue that is particularly subject to the human rights doctrine of the margin of appreciation, Crown Dependencies must ensure that Convention rights are not violated.^26^ This constitutional obligation is generally known as the responsibility of ‘good governance’.^27^ The Ministry of Justice then advises the Privy Council as to whether the Monarch should make an Assenting Order.

An interesting difference to note is that none of the Crown Dependencies operate a party system as is common across the UK. Elected representatives run on individual manifestos developed in line with their own views and priorities. There are some examples of individual representatives grouping together to form quasi-parties, but the systems of governance are not setup to be party political.

The full picture of the constitutional relationship between the Crown Dependencies is, of course, far more complex. There are also features of the political landscape in the Crown Dependencies that differ from Westminster, such as Guernsey’s committee system over ministerial cabinet. Nonetheless, the brief outline provided here is sufficient for our purposes.

## III. Abortion law in the UK and the Crown Dependencies

Before considering how the Crown Dependencies have approached the regulation of abortion, an overview of UK abortion law is needed. This is to enable the identification of points on which the Crown Dependencies might be said to have diverged from and, depending on one’s position on abortion, progressed further than the UK. It is also helpful to highlight the variation *within* the UK, contributing to an overall understanding of abortion law in the British Islands as rather a complex web.

In this section, we explore the regulatory approach to several key aspects of abortion law. These include gestational limits, grounds for termination, (de)criminalisation, the nature of healthcare professionals’ roles in care provision, conscientious objection, and location of care restrictions, allowing for a true picture of the regulation of abortion. Whilst we do identify points of alignment and convergence between the jurisdictions, a comprehensive comparative analysis is beyond the scope of this work. We hope that future scholarship will delve further into some of these individual considerations.

### A. United Kingdom

The UK is not a single jurisdiction where abortion law is concerned. This is a devolved policy matter, so although there is significant similarity between some of the UK nations—constituting a ‘wholly distinctive regulatory regime’^28^ that is often referred to as abortion exceptionalism^29^—there are ultimately four separate regulatory regimes.^30^

In England and Wales, termination of pregnancy is a criminal offence under the Offences Against the Person Act 1861 (OAPA 1861) and Infant Life (Preservation) Act 1929 (ILPA 1929), although, as we discuss below, people who end their own pregnancies have recently been excluded from criminal liability. The latter incorporates the born alive threshold, creating the crime of ‘child destruction’ where one intentionally causes the death of a ‘child capable of being born alive’.^31^ In 1939, *R v Bourne* established that some instances of termination must be lawful given the use of the word ‘unlawful’ in the OAPA 1861,^32^ setting the scene for legislative clarification (and progress) in the 1960s.

A significant change came with the Abortion Act 1967 (AA 1967). Whilst not decriminalising abortion, the AA 1967—which remains in force—has substantially improved access to lawful termination. It stipulates grounds on which abortion may be provided without prosecution, effectively acting as defences^33^:

- To save the pregnant person’s life (Ground A)^34^;
- To prevent grave permanent injury to the pregnant person’s physical or mental health (Ground B)^35^;
- In the first 24 weeks’ gestation, to prevent damaging the pregnant person’s physical or mental health when compared with termination (Ground C)^36^;
- In the first 24 weeks’ gestation, to prevent damaging the physical or mental health of the pregnant person’s existing children when compared with termination (Ground D)^37^; and
- Where there is a substantial risk of the child being born with a serious handicap (Ground E).^38^

These grounds do not, on the face of them, permit abortion on demand. Nonetheless, what has become known as the ‘social ground’ (Ground C) is broadly interpreted, accounting for 98 per cent of terminations.^39^

Grounds aside, the AA 1967 provides that no person has a duty to participate where they have a conscientious objection unless it is necessary to save life or prevent grave permanent injury.^40^ The boundaries of this right were established in *Greater Glasgow Health Board v Doogan*, such that ‘participate’ means doing so in a ‘hands-on capacity’ rather than there being a right to refuse to perform ancillary administrative or managerial tasks.^41^

Further change has come in recent years. Home use of misoprostol for early medical abortion was permitted by both nations in 2018.^42^ This came from a recognition that requiring this to take place in an approved clinical environment served no medical purpose whilst causing traumatic experiences for those who, for example, began to miscarry whilst on their journey home. Then, amidst the COVID-19 pandemic in 2020, home use was extended to the first drug used in an early medical abortion—mifepristone—as well as it being permitted for consultations to take place remotely.^43^ Initially introduced as a pandemic response, these provisions were made permanent under the Health and Care Act 2022.^44^

Importantly, the scope of criminalisation has recently been reduced, with a person who ends their own pregnancy within the lawful parameters of the AA 1967 no longer committing an offence.^45^ The impetus for partial decriminalisation in England and Wales is multi-factorial and may be seen as the culmination of a long campaign coupled with political willingness to tackle this issue. Nonetheless, the steep rise in prosecutions was undoubtedly an important factor.^46^ This prompted the Royal College of Obstetricians and Gynaecologists and others to intervene with guidance imploring professionals not to contact police if they suspected an offence.^47^ It is also likely that decriminalisation in the Crown Dependencies (discussed in subsequent sections) might have presented a further nudge towards partial decriminalisation in England and Wales.

The Crime and Policing Act 2026, however, does not apply to Scotland,^48^ where abortion remains a criminal offence, albeit by common law rather than statute.^49^ The AA 1967 extends to Scotland,^50^ thus making termination lawful within certain parameters. Both home use^51^ and telemedicine^52^ were introduced in Scotland within similar timeframes (indeed, Scotland was the first mover on home use). The same conscientious objection provisions apply, with the *Doogan* case having originated in Scotland. Finally, to round things off, the question of decriminalisation has also recently been raised in Scotland as part of a broader abortion law review.^53^

Northern Ireland is where variation is most apparent. Owing to several factors—including historical context and political instability—Northern Ireland had long been viewed as lagging behind Great Britain on abortion law. Before 2019, it had the most restrictive system in the UK, with access so limited that residents frequently travelled to Great Britain to access care.^54^

The OAPA 1861 applies in Northern Ireland, supplemented by the Criminal Justice (Northern Ireland) Act 1945 in a fashion mirroring the ILPA 1929 in England and Wales. However, the AA 1967 does not extend to Northern Ireland.^55^ The common law defence from *Bourne* is applicable in Northern Ireland for more serious eventualities but has long been more restrictively interpreted by Northern Irish judges.^56^

Progress came in Northern Ireland, somewhat controversially, through Westminster intervention. Abortion remained heavily restricted until 2019 when, following the failure of the Northern Ireland Assembly to re-form, Westminster passed the Northern Ireland (Executive Formation, etc) Act 2019. Amongst other things, this acted to (partially) decriminalise abortion in Northern Ireland by repealing the relevant sections of the OAPA 1861 in Northern Ireland.^57^ However, it remains an offence for anyone other than the pregnant person themselves to terminate a pregnancy that satisfies the born alive threshold.^58^ Supplementary regulations also acted to permit home use of misoprostol (but not mifepristone).^59^ We are thus in a somewhat strange situation whereby Northern Ireland has, in some ways, the most progressive abortion regulation of the UK (i.e., partial decriminalisation) whilst, in other ways, the most restrictive (i.e., home use is only permissible for misoprostol). This is all the stranger for this situation being created by Westminster, with the UK Parliament imposing partial decriminalisation on Northern Ireland without passing comparable legislation closer to home.

What we hope to have demonstrated here is that despite devolution there is significant similarity in abortion regulation across Great Britain and, to a lesser extent, the whole UK. There is much to be said for processes of policy transfer and divergence within UK abortion regulation; however, such discussion is beyond the scope of this paper because our focus is on developments in the Crown Dependencies.

### B. Isle of Man

Significant changes to the Isle of Man’s abortion regulation came with the Abortion Reform Act 2019 (ARA 2019). Prior to this, much like in the UK (except for Scotland), termination of pregnancy was committed to the statute books as a criminal offence during Victorian times. Whilst England, Wales, and Northern Ireland have the OAPA 1861, the Isle of Man has its Criminal Code 1872. The now repealed sections 71 and 72 criminalised the pregnant person who acts to end their own pregnancy,^60^ anyone assisting,^61^ and anyone supplying or procuring the means to perform an abortion.^62^ Interestingly, there was a distinction between the seriousness of these crimes. Whereas the OAPA 1861 allows life imprisonment for the person acting to end their own pregnancy,^63^ the Isle of Man imposed a limit of two years’ imprisonment.^64^ The person assisting, however, faced life imprisonment.^65^ This demonstrates a longer-term acknowledgement on the Isle of Man that the degree of seriousness, as far as the criminal law ought to be concerned, is variable between the parties in the abortion context. As we will come to discuss, this distinction has been maintained—albeit in a different formulation—in the recent reforms. It is also a pattern that is more commonly observable in recent years, such as in Northern Ireland,^66^ as part of the recent push for partial decriminalisation in England and Wales,^67^ and a matter of consideration in Scotland.^68^

The Code was supplemented by the Infanticide and Infant Life (Preservation) Act 1938 (IILPA 1938). Similar to the ILPA 1929, the IILPA 1938 applies a born alive threshold in defining child destruction.^69^ For the avoidance of doubt, the IILPA 1938 is clear that 28 weeks’ gestation is considered prima facie proof that this threshold is met.^70^ Further, it provides that where the termination is done in good faith to preserve the pregnant person’s life, there is no crime.^71^

With the passing of the AA 1967, the Isle of Man did not move to replicate it. Rather, almost three decades later, the Termination of Pregnancy (Medical Defences) Act 1995 (ToPMD 1995) was passed. The ToPMD 1995 has echoes of the AA 1967 but is not a copy.

The ToPMD 1995 provided a defence for a surgeon terminating a pregnancy provided certain criteria were met. Indeed, it has been recognised that the 1995 Act ‘was based on legal arguments to protect doctors, rather than a moral argument to protect women’.^72^ Varying slightly from the AA 1967’s two-doctor model, the ToPMD 1995 is specific in requiring that the termination is, in good faith, deemed to be ‘necessary to preserve the life of the pregnant woman’ by the surgeon who will carry out the termination and an independent medical practitioner.^73^ This differs from the AA 1967 in two ways. First, one of the two professionals must be the one carrying out the termination. The AA 1967 requires that two medical practitioners are satisfied that the termination meets the criteria but does not prevent a third professional carrying out the termination. The ToPMD 1995 also more strongly implies that the termination be surgical in referring to the termination ‘by a hospital surgeon’.^74^ Elsewhere, the greater precision as to healthcare professionals is apparent in that a termination justified by grave permanent injury to the pregnant person’s mental health must involve a consultant psychiatrist as the independent medical practitioner.^75^

Taking a stronger stance on the rights of the unborn child, the ToPMD 1995 contains a ‘[d]uty to preserve life of child’.^76^ That is, any termination after 24 weeks must be ‘best calculated to preserve the life of the child’.^77^ This section does clarify that the life of the pregnant person takes priority where applicable (e.g., life-threatening complications),^78^ but is still stronger than the AA 1967 in limiting the rights of the pregnant person.

In keeping with this, perhaps the starkest variation between the ToPMD 1995 and AA 1967 is that the former does not include the social ground. Under the ToPMD 1995, there is only a defence to an abortion in cases of: risk to life,^79^ to prevent grave permanent injury,^80^ foetal abnormality,^81^ rape,^82^ and incest.^83^ There is no provision for termination where pregnancy will pose merely a greater risk to the pregnant person’s mental health than termination—the threat to mental health must be of ‘grave permanent injury’.^84^ It is, of course, debatable when this standard might be met, but it is at least worth recognising that despite the ToPMD 1995 coming several decades after the AA 1967, the Isle of Man chose not to include the broader social ground. In introducing the Abortion Reform Bill, Dr Alex Allinson MHK (Member of the House of Keys) spoke of how in the debates surrounding the ToPMD 1995 when still a bill, the then House of Keys ‘rejected the British legislation [AA 1967] and any concept of what was termed ‘abortion on demand’’.^85^ It was, then, a deliberate choice to maintain a regulatory landscape overall more restrictive than the AA 1967.

For over 20 years, the ToPMD 1995 stood as the Isle of Man’s take on abortion regulation. This model of medical defences, much like Great Britain’s, retained the classification of abortion as a criminal offence, medicalising it to only permit termination where set criteria are met in the eyes of the healthcare profession. Introduced by Dr Alex Allinson MHK as a private members’ bill,^86^ the Abortion Reform Bill sought to bring sweeping changes. So significant that during debates it was queried whether it would present problems in getting Royal Assent ‘if the Bill is seen by Westminster to be a step too far’.^87^

The lack of a party-political system in the Isle of Man was arguably key to the possibility of this change. Allinson had included abortion law reform as a key point on his election manifesto, that being his *individual* manifesto rather than one of a party he represents. When bringing the private members’ bill, a level of endorsement was therefore demonstrable—at least from Allinson’s electoral area. This is different to when a private members’ bill is brought by a Member of Parliament in Westminster, as they would have been elected on the manifesto of their party.^88^ Private members’ bills are also more viable in the Isle of Man generally as there are not the same time restrictions on debate, which comes back to the fact that MHKs are individually elected.

Passed in 2019, the ARA 2019 has in some important ways gone further than any UK nation in liberalising abortion law. Most significantly, it repealed sections 71 and 72 of the Criminal Code 1872^89^ and the ToPMD 1995,^90^ thereby decriminalising the termination of pregnancy.^91^ Though this is limited to the pregnant person, with any other party acting to procure an abortion outside of the parameters of the ARA 2019 committing a criminal offence.^92^ As such, whilst the ARA 2019 still regulates abortion provision with the imposition of certain limits, provision outside of these stipulations no longer sees the pregnant person facing a custodial sentence.

As for the conditions for provision, during the first 14 weeks of gestation, abortion is available on request.^93^ That is, no criteria must be met, nor medical opinions sought; it is simply a matter of the pregnant person wishing to terminate their pregnancy. During this period, termination may be carried out medically.^94^ From the 15th week, there are criteria, further subdivided by gestation. During weeks 15 to 23 inclusive, an abortion may be provided on request only if:

- Continuing the pregnancy would pose ‘a substantial risk of serious injury’ to the life or health of the pregnant person^95^;
- There is a substantial risk of the foetus having a physical or mental impairment that would either be debilitating after birth or would result in death in utero^96^;
- The pregnancy resulted from rape, incest, or other unlawful intercourse^97^; or
- The termination is justified on ‘serious social grounds’.^98^

The final two options are to be determined ‘according to the woman’. Whilst there is no case law to test this,^99^ one assumes anyone seeking an abortion would be taken at their word. As for what constitutes ‘serious social grounds’, the Act provides the definition:

circumstances or conditions affecting the woman which are long-term and will have, or can reasonably be expected to have, a significant, adverse and enduring impact on her health.^100^

Termination from week 24 onwards is more restricted. Per the ARA 2019, a registered medical practitioner may provide an abortion from 24 weeks if they are of the opinion, formed in good faith, that it is appropriate in line with one of five justifications:

- Prevention of ‘grave long-term injury’^101^;
- Where the risk to life is higher if continuing the pregnancy^102^;
- Substantial risk of the foetus dying before or during labour^103^;
- Severe foetal developmental impairment that means the child would die shortly after birth^104^; or
- The child would suffer a ‘serious impairment’ likely to be limiting.^105^

The ARA 2019, then, follows the familiar pattern of restrictions on access to abortion care increasing with gestation. What sets it apart—particularly from the AA 1967—is that early in pregnancy there are effectively no restrictions. Abortion being permitted on demand, albeit restricted to the first 14 weeks, was a major step by the Isle of Man.

An interesting provision of the ARA 2019 is its clear statement that termination is not permitted based on gender.^106^ This applies across sections 6 and 11 of the Act, encompassing termination at any point in pregnancy. The exclusion of gender-based decisions may be a feasible stipulation from week 15 when the abovementioned criteria apply. What is unclear, however, is how it applies to terminations in the first 14 weeks. All that is needed to permit abortion in the first 14 weeks is the request of the pregnant person^107^—the Act does not require that any particular reasoning be provided. Whilst healthcare professionals providing abortion care would be expected to obtain valid consent^108^ in line with good medical practice, that would not necessitate the pregnant person providing a specific reason for wanting an abortion. This provision concerning the foetus’ gender, then, cannot feasibly be applied consistently to terminations in the first 14 weeks of gestation, making it partially redundant.

As is somewhat common where abortion provision is concerned, the ARA 2019 allows healthcare professionals to conscientiously object to participation in such care.^109^ Subject to the requirement to provide treatment to save the pregnant person’s life or prevent grave permanent injury,^110^ healthcare professionals may refer an abortion seeker to another professional if they object.^111^ Further reinforcing the importance of conscientious objection not causing serious harm or death, a professional who fails to act in accordance with these stipulations commits an offence subject to a fine or up to two years in custody.^112^ There is, then, further similarity with the UK wherein conscientious objection is permitted provided there is no risk of death or serious harm.^113^

Regarding healthcare professionals, though, the ARA 2019 is more progressive than its neighbours in dispensing with the requirement for two doctors to certify a termination, instead requiring just one.^114^ Whilst this may not immediately seem such a drastic change, testament to its significance is Lord Brennan’s questioning of it when appearing before the House of Keys:

If it has been good for the last 50 or 60 years in England and Wales and here for the last 20-odd years, why the change?.^115^

For all its more progressive features regarding access to abortion care, the ARA 2019 does stipulate that the Department of Health and Social Care (of the Isle of Man) ‘must secure the provision of appropriate counselling and support’ for anyone seeking to terminate their pregnancy.^116^ That said, such counselling is to be *offered* to abortion seekers but is not compulsory,^117^ and guidelines must ensure balance and impartiality.^118^ It is not, then, as strict as the much criticised German approach of counselling to ‘protect the unborn life’.^119^

Part 3 of the ARA 2019 goes beyond abortion provision to include access zones. The ARA 2019 empowers the Department of Health and Social Care to create access zones around premises providing abortion care^120^—including both terminations and associated counselling—as well as the homes of healthcare professionals involved in abortion care.^121^ The inclusion of healthcare professionals’ homes is a step further than was later implemented in Northern Ireland,^122^ England and Wales,^123^ and Scotland.^124^ Arguably, this is of greater necessity in the Isle of Man given the population size and thus likelihood of people knowing the addresses of relevant healthcare professionals.

Within access zones, individuals are prohibited from acting ‘with the intention of dissuading anyone’ from providing or accessing services or harassing or intimidating a provider or service user.^125^ They must also not photograph, record, or sketch any provider or service user within an access zone.^126^ Doing any of these things after being warned by the police not to constitute a summary offence with a penalty of up to 12 months in custody or a fine of £10,000.^127^

Interestingly, the ARA 2019 provides the defence to breaching an access zone of being a patient seeking or receiving abortion services.^128^ Whilst this may be understandable for the possibility that someone accessing care is mistakenly identified as breaching an access zone, it does create an unusual, albeit unlikely, possibility whereby someone accessing abortion services would be permitted to protest and discourage others from accessing abortion. This may seem hypocritical, but it could happen, as we know that some who access or have accessed abortion services nevertheless consider themselves anti-abortion and may even be activists, such as Abby Johnson.^129^

As an aside, it is worth acknowledging that the creation of access zones for abortion services has prompted the Isle of Man to consider extending such protections to a whole range of health and social care facilities. The Safe Access Zones and Protected Public Places Bill 2024 is currently before Tynwald. Dr Michelle Haywood MHK, who introduced the private members’ bill, acknowledged that it ‘aims to, in effect, extend the similar access protection that was put in place as part of the [ARA 2019] to apply to other health and social care settings’.^130^ Whilst this development has seen opposition,^131^ it is unusual for abortion law to prompt such a broad change to the health and social care landscape.

Whilst not necessarily a critique, it is, for completeness, worth acknowledging that the ARA 2019 has no provisions relating to the use of telemedicine in abortion care. This might be considered a shortcoming by some given the growing trend to improve access in this way. However, it is important to remember the small island context. Arguments for telemedical abortion often focus on access for those who may struggle to attend in-person appointments, especially those who live rurally. This is far less of a concern on the Isle of Man (and, indeed, all the Crown Dependencies). The size of the island—33 miles in length—means it generally takes little over an hour to drive from one end to the other. With the capital of Douglas being relatively central, even those living at either end of the island are not far. Of course, permitting telemedical care provision would have been yet more progressive and would have provided additional support for some especially vulnerable individuals who may, for example, be victims of coercive control^132^ or, in the case of younger people, may find it hard to seek services without parental knowledge.^133^ However, the extremely limited need for abortion services in the small island context alongside the fact that the ARA 2019 came before the wider shift to telemedicine in the UK at least makes this omission understandable, even if less than ideal.

### C. Guernsey

Abortion was not historically criminalised in Guernsey. It was not until 1910, when it was recognised that there was ‘no law in force in the Island of Guernsey for the punishment of persons attempting to procure abortion’,^134^ that the States passed the Loi sur L’Avortement.^135^ Article 1 of this law criminalised any woman acting to end her own pregnancy and any person acting to end the pregnancy of someone else, with potential for a sentence of life in penal servitude.^136^

This law stood for 86 years until Guernsey became ‘the last place in the British Isles to legalise abortion’ with the passing of its Abortion (Guernsey) Law 1997 (AGL 1997).^137^ Coming shortly after the Isle of Man’s ToPMD 1995, the AGL 1997 set out circumstances in which a termination would be lawful. This was a response to recognition that, at the time, roughly 150 women travelled to the UK each year to access abortion care,^138^ returning with no legal action. The 1910 law, then, was presenting more of an inconvenience than a deterrent or the originally intended punishment.^139^

The AGL 1997 begins by clarifying the criminalisation of any woman who either administers herself ‘any poison or other noxious thing’^140^ or uses an instrument or other means^141^ with the intent of procuring a miscarriage. Similarly, any other person who, by the same means, seeks to procure a woman’s miscarriage is confirmed as guilty of an offence.^142^ Aligning more with England and Wales at the time, both offences were subject to life imprisonment.^143^ That is, there was not the distinction made by the Isle of Man, which stipulated a lesser sentence for the woman seeking to procure her own miscarriage.^144^ There was, however, a lesser sentence—not exceeding three years’ imprisonment—for anyone guilty of supplying the means to procure a miscarriage.^145^

Further to this, the AGL 1997 provided an exception to criminalisation in set circumstances. The two-doctor, good faith requirement is present,^146^ with termination permitted where there is a risk to life,^147^ to prevent grave permanent injury,^148^ or where there is a substantial risk of foetal abnormality (up to 24 weeks).^149^

Unlike the Isle of Man’s ToPMD 1995, the AGL 1997 *does* include a social ground. That is, termination is permitted within the first 12 weeks of pregnancy if ‘the continuance of the pregnancy would involve risk, greater than if the pregnancy were termination, of injury to the physical or mental health of the pregnant woman or any existing children of her family’.^150^ There are echoes of the AA 1967 here. Indeed, as in England and Wales, this provision came to be interpreted broadly in Guernsey, accounting for 93 per cent of terminations in the period 2020 to 2022.^151^ In practical terms, then, for the last few decades abortion has been widely permissible in Guernsey when sought early in pregnancy.

Notably absent is an explicit ground based on rape or incest. However, this might be thought to be covered by the inclusion of a social ground. Indeed, the 12-week limit on the AGL 1997’s social ground does align temporally with the ToPMD 1995’s rape/incest ground.^152^ Guernsey’s approach here is more in keeping with the AA 1967, wherein there is no explicit mention of rape nor incest as grounds for abortion.

A significant update to the regulation of abortion care in Guernsey recently came in the form of the Abortion (Guernsey) (Amendment) Law 2021 (AGAL 2021). As noted by the Explanatory Memorandum, the new law exists to ‘abolish the offence of a woman procuring her own miscarriage’.^153^ Decriminalisation, then, was very much at the core of this effort, there being a recognition that Guernsey’s law concerning abortion was, at the time, ‘more restrictive than other medical procedures and compared with other jurisdictions’.^154^ Indeed, this comparative consideration was prominent in the development of the new law. The Committee for Health & Social Care was clear that despite the clear focus on modernising Guernsey’s abortion law, it was ‘not presently suggesting to reproduce the Isle of Man approach at this time’.^155^ Early in the legislative process, the planned changes in Guernsey received strong (though not unanimous) public support, with many finding the law to be outdated and unnecessarily restrictive of access to care.^156^

So, what does Guernsey’s attempt to ‘future proof’^157^ look like? The AGAL 2021 does not break things down to the extent of the Isle of Man’s ARA 2019. In acting to amend the AGL 1997, it is less of a sweeping reframing of the regulatory landscape. Whilst it incorporates significant changes, these are achieved within the existing framework to some extent.

The AGAL 2021 starts with the repeal of the section that criminalised the person seeking to terminate their own pregnancy.^158^ Indeed, the Committee for Health & Social Care made clear that ‘providing unregulated abortion services would remain illegal, but the women who use those services would not themselves be subject to criminal penalties’.^159^ As such, offences pertaining to the administration of any poison or other noxious thing or use of any instrument with intent to procure a miscarriage remain as applicable to other parties^160^—as does supply^161^—with the decriminalisation being solely in relation to the pregnant person.

Changes were also made to the roles of healthcare professionals in abortion care. Removing the long-standing two-doctor requirement, the AGAL 2021 has streamlined this to require just one recognised medical practitioner to form a good faith judgement.^162^ This came about from a recognition that a second practitioner was ‘an unnecessary step that may prevent a woman receiving the care she requires and may not be affordable for all’.^163^ Access, then, was seemingly a guiding principle. There is also greater recognition of the interdisciplinary team in the delivery of healthcare—and abortion care more specifically—in expanding the scope of healthcare professionals to include registered nurses and midwives.^164^

Further relating to the role of healthcare professionals in abortion care are amendments to conscientious objection rules. Whereas conscientious objectors were previously only required to provide care to save the life of the pregnant person, this exception now extends to the prevention of ‘serious injury to the physical or mental health’ of the person.^165^ Further, conscientious objectors are now required to inform the pregnant person of their right to see another professional and ensure they have sufficient information to exercise this right.^166^ In line with many other jurisdictions, this will limit the direct impact conscientious objection can have on individual abortion seekers. There is also an interesting addition of a right for the Committee to make regulations that further limit or restrict the exercise of conscientious objection.^167^ Whilst no such actions have currently been taken by the Committee, it will be interesting to see what further limits or restrictions might look like if ever introduced.

Finally, disparities between gestational thresholds in Guernsey as compared with Great Britain and the Isle of Man were acknowledged,^168^ leading to two notable increases. First, the 24-week limit on termination for reason of substantial risk of foetal abnormality was lifted,^169^ making abortion on this basis lawful up to term. Secondly, the 12-week limit for the social ground was doubled to 24 weeks.^170^ With these changes, access to care within the regulated healthcare system will be broadened alongside the more principled move to decriminalise terminating one’s own pregnancy.

As in the Isle of Man, Guernsey has made no provision for telemedical abortion care. Whereas—as acknowledged above—the Isle of Man changed its abortion law before the COVID-19 pandemic saw widespread uptake of telemedical care, this is not true of Guernsey. It might be said, then, to be a more active choice in Guernsey *not* to permit telemedical abortion care which, as we have already pointed out in relation to the Isle of Man, may detrimentally impact especially vulnerable groups.

### D. Jersey

Our discussion of Jersey will be slightly different in approach for the reason that Jersey is amidst a process of significant regulatory change concerning abortion care. As such, we outline both the current (and very dated) framework that continues to apply, as well as the new system that will replace it in the near future.

Before the Termination of Pregnancy (Jersey) Law 1997 (ToPJL 1997) was passed, abortion was lawful under Jersey custom only for the purpose of preserving the life of the pregnant person, or if the continuance of the pregnancy would make them a ‘physical or mental wreck’.^171^ Outside those customary parameters, terminating a pregnancy would invite prosecution for murder or manslaughter, with an approach that diverged from the *rerum natura* (in existence, construed to mean ‘born alive’) requirement for homicide under English law.^172^ Before 1997, approximately 300 people per year travelled to England to access lawful abortion services.^173^

The ToPJL 1997 codified and amended the customary law to allow abortion by providing a statutory basis for the circumstances in which termination is not unlawful, with distinctions based on gestation. At any point in pregnancy, termination is permitted to save the life of or prevent grave permanent injury to the physical or mental health of the pregnant person.^174^ Up to the 24th week of pregnancy, termination is permitted where there is a substantial risk of the child being born with such physical or mental abnormalities as to be severely handicapped.^175^ Finally, and most restrictive, is lawful termination on the basis of the pregnancy causing distress to the pregnant person—permitted only during the first 12 weeks of pregnancy.^176^

Like the AA 1967, two doctors (‘registered medical practitioners’) are gatekeepers to lawful abortion. Distinctively, however, the ToPJL 1997 demands that one of the doctors must practise in obstetrics and gynaecology and the other must either practise in an area relevant to the pregnant person’s medical condition (where terminating under the first of the above grounds)^177^ or be a paediatrician (where terminating under the second of the above grounds).^178^ This can be seen as more restrictive than the more common formulation of the two-doctor requirement, as it is reliant on the availability and agreement of a more limited pool of professionals.

Conscientious objection is protected, such that no person is under a duty to participate in ‘treatment’ except where it is necessary to save a life or prevent grave permanent injury.^179^ This approach echoes the AA 1967 and the other Crown Dependencies (and, indeed, many other jurisdictions internationally). Combining this right to conscientious objection with the abovementioned requirements of the ToPJL 1997 regarding the specific job roles of the registered medical practitioners involved in abortion care does present a challenge to care delivery in a small island setting, further reducing the pool of potential providers of termination.^180^

Regarding the possibility of telemedical care delivery, Jersey abortion law has persisted in requiring face-to-face consultation. ‘Consult’ is expressly interpreted to mean ‘consult in person’.^181^ The ToPJL 1997 also demands that termination is only lawful, except in an emergency, when carried out in an ‘approved place’, which means a hospital or a ‘nursing home’.^182^ In practice, the only place approved is the Jersey General Hospital, so consultations must take place there, and for early medical abortions, the first dose of the abortion drug must be taken at the hospital.

Jersey’s latest annual report on the termination of pregnancy provides an insight into the reality of provision under this framework.^183^ Between 2023 and 2024, the number of abortions provided on the island increased from 260 to 280, with fewer than five terminations involving Jersey residents travelling to England and Wales. Early medical abortions account for 92 per cent of terminations, thereby falling within the most flexible lawful basis for abortion within the first 12 weeks. Recalling that before the 1997 law approximately 300 people per year travelled to England for an abortion, we see that access to abortion on the island has seemingly improved significantly. Nevertheless, in 2023, the Government committed to review abortion law and access in Jersey.

The liberalising reforms in the other Crown Dependencies might have influenced this review. Similarly, the fact that abortion is comparatively freely available up to 24 weeks of pregnancy in England, Wales, and Scotland would presumably have exerted some pressure on attitudes towards the stricter 12-week limitation of the ToPJL 1997. The background section to the draft law published in January 2026, however, makes it clear that Jersey views the AA 1967 as anachronistic due to the specific, limited grounds upon which termination may be justified, stating that the AA 1967 ‘had not been updated in accordance with the provisions of other jurisdictions and changing practice’.^184^ With the proposed changes currently existing only as a draft law, we will consider both the consultation process and the resulting proposals, recognising that none are in force at the time of writing.

Jersey’s propensity for consulting the public on significant legal questions led to important findings concerning abortion, reported in early 2024.^185^ There was overwhelming support for the availability of abortion where there is either a risk to life (95 per cent) or grave risk to health (91 per cent). Support for abortion on request (that is, without the requirement for justification) was also surprisingly high at 74 per cent. A small majority of 59 per cent felt that gestational limits should depend on the reason for termination, with most agreeing there should be no gestational limit for abortion for reasons of risk to life, grave risk to health, foetal abnormality, rape, or incest. As for the question of criminalisation, just 20 per cent of respondents felt that pregnant people should be subject to criminal prosecution for termination outside of the legal framework.

After the public consultation, the Government published its initial proposals for reform in March 2025,^186^ followed by publication of the draft law in January 2026,^187^ which was then passed in March 2026.^188^ The new law will permit termination up to 21^+6^ weeks on any grounds,^189^ meaning the existing justifications will be swept away and there will be ‘no requirement in law for the woman to have a reason/state the reason why she is having a termination’.^190^ This significant shift from the 1997 Jersey law is intended to promote autonomy and reduce stigma, and it aligns with the World Health Organization position ‘that termination should be available without justification of need’.^191^ It is noted that the 21^+6^ week time limit relates to viability, and the proposal states that ‘terminations for non-medical grounds should be one week before the end of 22 weeks gestation, which international consensus states is the “cut off” for viability of life’.^192^ Another important change is that a woman will only need to consult one doctor (rather than two under current law) for a termination before 22 weeks.^193^

At later gestations, termination by a registered medical practitioner will be permissible under specific grounds. Largely in line with other jurisdictions, these grounds include: to save the pregnant person’s life; to save another foetus; to prevent significant injury to the pregnant person’s physical or mental health; or where there is a significant risk of serious foetal abnormality.^194^ Whilst it will remain necessary to consult two doctors, only one consultation will have to be in person,^195^ and the limitations of the outgoing law (requiring specialist participation) will also be removed, thus enabling a wider pool of doctors to provide the service. The right to refuse to participate will be safeguarded except when participation is necessary in an emergency.^196^ It was acknowledged by the Assistant Minister for Health and Social Services that only a limited number of doctors in Jersey are willing to provide terminations, which can have a service-limiting impact.^197^ Nonetheless, it was deemed important to protect this right of professionals.

Despite this liberalising shift, it is not envisaged that later-term terminations will be routinely available in Jersey. The potential gap between what is proposed in the law and what is feasible is noted by the Government:

Jersey has a small health care workforce when compared to other jurisdictions and, given workforce constraints and the need to provide safe services, it is unlikely that we will be able to provide terminations post 12-week gestation in Jersey at the point at which the draft Law comes into force.^198^

On this issue, campaigners have raised concern that people who are therefore prevented from accessing abortion in Jersey will be forced to travel to the UK at their own personal expense, unless the termination is deemed to be ‘medically necessary’ in terms of an urgent procedure needed to save a woman’s life.^199^ This became a point of (albeit minor) debate during the passage of the draft law. Noting that those in abusive relationships may not be able to travel to the UK for care at a later gestation, Deputy Tadier commented:

What we are hearing is this is a great thing to do just from a theoretical point of view, but I am interested in the practical implications of what we are going to see here.^200^

Whilst many acknowledged this would be an issue for some, it was highlighted as something of an inevitability in the context of an island like Jersey. That is, with the small number of later-term terminations a professional would be carrying out, there would be a serious risk of deskilling.^201^ The Assistant Minister for Health and Social Services thus noted the choice to nonetheless make such care lawful to ‘destigmatise termination’.^202^ There is also an interesting question to consider where a Jersey resident travelling to England to access care will, as the law currently stands, be subject to greater restriction under the AA 1967. Potentially, then, this destigmatisation is even more symbolic when considering the reality of how care will be accessed by those unable to undergo a termination on the island.

Whilst only registered medical practitioners (doctors) will initially be permitted to provide terminations, Article 14 does allow for future Regulations or Orders—by the States and Minister, respectively—to permit other healthcare professionals to do so.^203^ This is possible for medical terminations up to a fixed gestation, meaning only doctors can provide surgical abortion. Nonetheless, given that the latest Jersey data show that 93 per cent of terminations are early medical abortions, there is the potential to significantly open up access for the majority of those seeking terminations—particularly in light of the abovementioned conscientious objection challenges. That is, of course, if the States or Minister choose to exercise this power.

The new law will not, however, relax the requirement for in-person consultation, at least in the short term. Importantly, the power for the Health Minister to make an Order to do so is included, either in ‘extraordinary circumstances’, such as a pandemic,^204^ or if the Assembly determines it is appropriate, for example, ‘in response to increased societal acceptance’.^205^ Nonetheless, it was determined that telemedical care will not be brought in ‘as the same geographical access issues do not exist in Jersey’.^206^ We will later explore the issues with such a view, as telemedicine is beneficial not only for reasons of geography.

The new law will remove the threat of criminal liability that is currently present for women straying outside the provisions of the 1997 law, stating that a woman who either knowingly consents to a termination outside the law, or performs or attempts to perform a termination outside the lawful provisions does not commit an offence.^207^ The risk of criminal liability is reserved for those who knowingly perform or are involved in terminating a pregnancy of another.^208^

The UK Parliament has recently decriminalised abortion for people who terminate their pregnancy outside the lawful parameters of the AA 1967.^209^ However, in 2023, when the Jersey review began, decriminalisation in the UK (except Northern Ireland) was not on the horizon. Indeed, the most significant development in the UK Parliament until very recently has been the move to permanently allow remote access, which, as we have noted, appears not to have been influential in Jersey.

The most controversial issue for Jersey politicians during debate was the charging of fees. Context is important, in that healthcare is not provided through a UK-style National Health Service model in Jersey. Even so, concern was raised that cost may present a barrier for some, particularly where the reason for seeking a termination is a lack of financial means to raise a child.^210^ The Attorney General did clarify that the provision on fees is ‘permissive, not mandatory’.^211^ Nonetheless, this matter resulted in Article 17—that containing provision for fees—was the only article subject to a separate vote, passing only by a narrow margin.^212^

This new law evidently presents a more principled approach than the highly restrictive 1997 law, but we also note some arguably inconsistent features—inconsistent in spirit, at least in terms of how Jersey has and has not chosen to liberalise its law. On the one hand, there is a clear commitment to promoting autonomy by allowing termination before 22 weeks without requiring a justification. This de-medicalises abortion by removing potential obstacles to access as well as extending the time limits for access (at least in principle, acknowledging the practical reason why terminations beyond 12 weeks are not possible except in emergency). However, as was highlighted in debates, this 22-week limit is lower than that in many other jurisdictions.^213^ The AA 1967, for example, imposes a 24-week limit on certain grounds for termination. There is a balance to be struck here as to which is preferable. An additional two weeks are permitted in Great Britain, but the termination must satisfy one of the statutory grounds. The limit is lower in Jersey, but during those 22 weeks no justification is required.

Furthermore, the new law will not automatically permit the use of telemedicine in abortion care pathways despite it now being common practice (including across the UK). It is being acknowledged, then, that we are in an era of telemedical abortion care whilst stopping short of incorporating it. The reasons given for not allowing remote access are that on a small island the geographical ‘practical difficulties’ present in the UK are not a barrier to access, and concern expressed by professionals that women may be either unaware of the true stage of pregnancy or may conceal the truth.^214^ The first point has some traction; like the Isle of Man and Guernsey, Jersey is small and there is a reasonably good bus service.^215^ However, this overlooks the issue already highlighted in relation to the other Crown Dependencies; that some vulnerable people might struggle to access confidential treatment if they have a controlling partner or parent.^216^ The second reason reveals a professional bias towards assuming that pregnant people are routinely mistaken in understanding their stage of pregnancy or are likely to deceive. Thus, on a very practical aspect that could make a difference to those seeking abortion care, the law will remain restrictive whilst at the same time, the law is liberalised in a way that cannot be realised on the ground.

An interesting observation of the very brief period of debate over the new law is the lack of proposed amendments even though deputies raised concerns over aspects of the draft law. On several occasions, deputies called for certain improvements, though commenting that they should be part of revisiting the legislation in the future. Seemingly, this was out of a desire to push through the legislation with a “better than what we currently have” attitude. Indeed, Deputy Doublet expressed concern over the application of viability in setting the gestational limit, though commented that ‘it is an improvement on the current legislation, and on that basis I would support it at this time’.^217^

Given this approach of flagging concerns to be addressed at a later date, the law passed very quickly indeed, and with very little opposition.^218^ As noted in the draft law, the new framework will come into force on 1 September 2026 or on an earlier date specified by the Minister by Order.^219^

## IV. Progressing beyond the UK?

What is apparent from our exploration of the approaches to abortion law in the Crown Dependencies is that they do not unreflectively follow the example of Westminster or, indeed, any of the devolved UK nations—at least not anymore. Certainly, there are clear parallels that we have observed. Many general concepts can be seen reflected across the UK and the Crown Dependencies, such as variation in access based on gestation. Nonetheless, it is not a straightforward matter of the Crown Dependencies following closely behind the UK in this area. Indeed, on the issue of decriminalisation, England and Wales may be seen to have followed the examples set by the Isle of Man and Guernsey (and, to a lesser extent, Jersey), thus illustrating a policy influence “push and pull” between the UK and the Crown Dependencies as closely related yet independent jurisdictions.

In many respects, this clear legislative independence has resulted in the Crown Dependencies lagging behind much of the UK in the more progressive approach to the regulation of abortion that has developed over the last 60 years. Despite having fallen behind for many years, more recently politicians in the Crown Dependencies have not been shy in criticising the framework of the AA 1967 amidst their recent liberalising reforms. This shift in attitude seen in all the Crown Dependencies, leading to the recent developments, may be viewed as test cases, or at least gentle nudges, to encourage further liberalisation of abortion law across the UK. The partial decriminalisation (for pregnant people) of abortion in England and Wales represents an important step towards a more progressive abortion law within the UK that aligns more closely with that of the Crown Dependencies (pending Royal Assent of the Jersey law).

Regardless of any Crown Dependency’s desire to update abortion regulation, it is worth acknowledging practical realities that are at least contributing factors to their limited capacity to do so. First is the simple matter of policy resources. The Crown Dependencies are markedly smaller jurisdictions than the UK—and any of the UK nations taken individually—so have comparatively small civil services.^220^ Smaller jurisdictions face similarly long lists of policy areas to address, but with fewer resources must inevitably adopt a stricter prioritisation approach. After all, it takes just as long to draft a bill whether it will affect 100,000 people or 10,000,000 people.

This relates to a second practical reality: that abortion care is arguably a less pressing issue in the Crown Dependencies. That is not to suggest that pregnant people seek abortions at any lesser rate on these islands. Rather, there are reasons unrelated to the law that these individuals will seek care *off island*.

One reason is a straightforward matter of resources that means surgical abortion is generally not available on the islands. Smaller populations mean smaller healthcare services, which in turn means that more specialised—or, indeed, less common—procedures often require people to travel to the UK for care. With surgical abortion being an example of this, later-term abortions cannot as easily be provided, and those preferring a surgical abortion at a lower gestational age may be forced to travel. This is not quite the same situation as has historically been the case with Northern Ireland, whereby pregnant people were forced to travel to England for terminations.^221^ Whilst for a long time Northern Ireland and the Crown Dependencies were in similar situations of the law preventing this care, it was at least feasible in Northern Ireland in terms of the size of its healthcare system. In the Crown Dependencies, regardless of the law, it would be challenging to justify significant surgical abortion provision due to how few people would be accessing it. It would require the recruitment of professionals with appropriate expertise who would then be at risk of deskilling due to how infrequently they would be performing the procedure (whereas abortion care providers in a larger jurisdiction will routinely be carrying it out, particularly in organisations such as the British Pregnancy Advisory Service). This reality is brought into focus by Jersey’s recent legislative change, with a Health and Community Services report making clear that under the new law:

There will be a difference between what is permitted in law and what is provided in Jersey because we do not have the facilities and skills required to safely deliver later stage terminations in Jersey.^222^

We have highlighted the concern that pregnant people compelled to travel to access services may be expected to pay rather than having a free service at home, but another reason for accessing abortion care in the UK is that of confidentiality. Or, more accurately, anonymity. Living on a small island entails a greater level of interconnectedness with one’s fellow citizens. Even with professionals upholding confidentiality, it is understandable that someone accessing abortion care would fear word spreading within their community. It is also very possible they will know the professional providing the service personally and have no choice to use an alternative provider as they would in a larger jurisdiction. Hence, it was acknowledged by the then Isle of Man Minister for Health and Social Care that in the initial years following the implementation of the ARA 2019, abortion care provided off island was ‘mainly as a direct result of confidentiality challenges’.^223^ Indeed, some of those working in health policy on the Crown Dependencies suspect that the number of people travelling to the UK for abortion care is higher than data suggest as a result of them not declaring their correct residency for the sake of privacy.^224^

Even in the small island context whereby no one will be a significant distance from an abortion provider, we might still question the approaches of the Crown Dependencies to exclude telemedical provision. At the time the Isle of Man changed its abortion law, telemedical provision was not available in Great Britain. Thus, whilst it was still available in various jurisdictions globally, it could at least be recognised as not the norm. Though it was clearly a more active choice to exclude telemedicine in Guernsey and Jersey. This is problematic as there may be pregnant people who wish to seek a termination but are in domestic situations that prevent them attending in-person appointments. We have noted this already, but it is deserving of further attention.

It is already understood that those who are, for example, victims of domestic abuse may face greater challenges in accessing in-person abortion care. If they do not want that abuser to know about the pregnancy (and intended termination) but their abuser exerts significant control over them, leaving their home for sufficient time to access care is not always feasible. This may be doubly challenging for such victims in the Crown Dependencies for similar confidentiality reasons to those already discussed. It will be far harder for a victim to avoid their abuser finding out they have attended an abortion clinic on a small island; it is more likely that they will be seen by someone they know who may then mention it to their abuser. There is not even the option of attending a clinic in, for example, a slightly further away city—that would mean travelling overseas.

What is more likely is these individuals finding time to undergo a remote consultation. Such a care pathway may even present an opportunity for the healthcare provider to identify a safeguarding concern that could help them get out of their abusive situation.^225^ This is especially relevant when one considers some of the reasoning deployed in the decision not to permit telemedicine in Jersey. The Assistant Minister for Health and Social Services noted that an insistence on in-person consultation was ‘to better support confirmation of gestational age or allow identification of potential coercion or abuse’.^226^ As the literature surrounding this has indicated, this position is problematic.^227^ For the reasons we have already discussed, victims of abuse may be better served by telemedical care provision.

Thus, the availability of telemedical care may enable access for those who would otherwise be forced into considering illegal (and potentially unsafe) means of termination. Even if they may be so few in number, abortion seekers in such domestic setups can be said to benefit even more from telemedical provision given the small island setting. As such, this is a matter on which the Crown Dependencies ought to consider aligning more closely with Great Britain, resulting in a more comprehensively progressive approach to abortion regulation.

There is no doubt that the Isle of Man’s 2019 changes represented both a significant departure from Westminster and a liberalisation of reproductive health regulation—to then be (partly) followed by Guernsey. Whilst Westminster may have acted to (partially) decriminalise abortion in Northern Ireland, until very recently there had been no move to match this progress closer to home.^228^ It is no surprise, then, that during the bill’s passage there were concerns raised as to the Isle of Man somewhat going it alone in departing from the UK:^229^

if this was legislation that brought the women of the Isle of Man up to the same level of support for abortion as they enjoy in the United Kingdom I am under no doubt that this will have been approved 24-nil [a unanimous vote in the House of Keys] a long time ago and we would have moved on. I think that is a key marker to put down. I support good change and I think everyone here does, so we are all agreed on this, but we are looking at legislation which goes significantly further and in some areas into totally uncharted waters.^230^

Even beyond the question of decriminalisation, various elements of liberalisation within the ARA 2019 have put it out of step with at least Great Britain. As acknowledged by Lord Brennan when he appeared before the House of Keys during the legislative process:

[The Isle of Man will] not just [be] catching up but leaving us behind. This is considerably more permissive legislation than we personally have in England and Wales. It will become known around our Islands and become a topic for public comment and debate, I have no doubt about that.^231^

Whilst they may not directly attribute motivation to the Crown Dependencies, that England and Wales have recently partly decriminalised abortion is likely to have been influenced by this progress, at least in part. Precedents created in neighbouring and closely linked jurisdictions (that share aspects of healthcare provision) are far more impactful than developments in other less connected jurisdictions. Certainly, decriminalisation has been at the core of some of the developments in the Crown Dependencies. Recall this being the fundamental purpose behind the reforms in Guernsey.^232^

Despite these more recent instances of liberalisation—or liberalisation in progress, in the case of Jersey—abortion care across the Crown Dependencies remains tightly regulated when compared with other areas of healthcare. This is something the three nations have in common with the UK and, indeed, many jurisdictions internationally. As such, whilst we suggest there are benefits to other nations looking to the Crown Dependencies for inspiration, there remains a need for reforms to move further forward in departure from abortion exceptionalism.

## V. Conclusion

Comparative abortion law is a significant area of scholarship but does not routinely incorporate developments in the Crown Dependencies. We have demonstrated that there are benefits to doing so because the three islands, which all share significant social, cultural, health, and legal links with the UK, provide important case studies, charting their own course towards increasingly progressive regulation rather than exclusively relying on the UK to take the lead. Historically, there has been a clear trend of the Crown Dependencies catching up with the UK. But recent years have seen a concerted and innovative shift towards going against the grain of Great Britain (recognising the differing situation in Northern Ireland) with an increasingly pioneering approach to health matters of the most morally sensitive nature. The examples discussed here in relation to the (partial) decriminalisation of abortion in the Crown Dependencies perfectly illustrate this shift.

Despite their collective title implying the Isle of Man, Guernsey, and Jersey’s dependence on the UK, we suggest that developments in abortion law demonstrate a “push and pull” between the UK and the Crown Dependencies, whereby the Crown Dependencies have traditionally lagged behind the UK but have recently embraced a far more pioneering attitude towards sensitive moral matters in health policy. Thus, the UK (and beyond) might benefit from looking the other direction once in a while and learning from the pioneering approaches that have recently emerged on these nearby islands. The Crown Dependencies’ insightful scrutiny of neighbouring legislation demonstrates how assessing the law in neighbouring jurisdictions with many crucial links can usefully inform progressive developments at home to avoid perpetuating problems seen elsewhere. This is especially timely given the recent decriminalisation of abortion in England and Wales and, indeed, debates over areas such as assisted dying, which has seen similarly pioneering reform in the Isle of Man^233^ and Jersey^234^ whilst progress has stalled in Westminster.^235^

## Footnotes

1 Our chosen ordering—Isle of Man, Guernsey, and Jersey—may be jarring to some readers as it is not the norm. We have opted for this order for purely chronological reasons relating to the most recent changes to abortion law, as will become apparent shortly.

2 Mental Capacity Law and Policy. 2024. *Assisted dying/suicide proposals in the United Kingdom and Surrounding Crown Dependencies: a Comparative Table* <https://www.mentalcapacitylawandpolicy.org.uk/assisted-dying-suicide-proposals-in-the-united-kingdom-and-surrounding-crown-dependencies/> accessed 25 May 2026.

3 Jordan A Parsons, *‘Opt Out’ Organ Donation: An Ethico-Legal Policy Analysis* (OUP 2025).

4 eg A recent, updated cooperation agreement was signed by the Isle of Man Government, Liverpool City Council, and Liverpool City Region Combined Authority. For geographical reasons, the Isle of Man’s healthcare relationship with the UK is largely concentrated in Liverpool and surrounding areas.

5 Independent Health and Social Care Review. 2019. *Independent Review of the Isle of Man Health and Social Care System* <https://www.gov.im/media/1365879/independent-health-and-social-care-review-final-report.pdfhttps://www.gov.im/media/1365879/independent-health-and-social-care-review-final-report.pdf>; Government of Jersey. 2022. *Review of Health and Community Services (HCS) Clinical Governance Arrangements within Secondary Care* <https://www.gov.je/SiteCollectionDocuments/Government%20and%20administration/Review%20of%20Health%20and%20Community%20Services%20Clinical%20Governance%20Arrangements%20within%20Secondary%20Care.pdf> accessed 25 May 2026.

6 This is a widely recognised phenomenon, though clear data are not available. This is because demographic reporting ordinarily groups those from the Crown Dependencies and the UK.

7 Of note, Guernsey and Jersey are geographically closer to France and have significant French influence historically, as is apparent in much of their formal legal and political language being French.

8 Until recently, this post was held by Paul Richardson. The interim Chief Officer is Professor Tim O’Neill. Both were recruited from the UK.

9 This is a dual post—both Director of Public Health and Chief Medical Officer. It is currently held by Dr Matt Tyrer. Incidentally, the Deputy Director—Dr Ross Keat—was also recruited from the UK.

10 And, indeed, with assisted dying legislation—though that is not our focus in this article.

11 s 241 Crime and Policing Act 2026.

12 eg in a debate in the Jersey States Assembly on 25 February 2026, the Acting Attorney General was questioned about this very issue. See States of Jersey Official Report, 25 February 2026, at 1030, per The Connétable of St. Lawrence.

13 sch 1 The Interpretation Act 1978.

14 The term Bailiwick simply means the area of jurisdiction, derived from the historical use of the word Bailiff when exercising the function of a Sheriff.

15
*R (Barclay) v Secretary of State for Justice (No 2)* [2014] UKSC 54, at para 6. See also Royal Commission on the Constitution, *Report of the Royal Commission on the Constitution 1969-1973 Volume I* (HMSO 1973), at para 1347.

16
*Barclay* (n 15). In *Barclay*, there is reference to the ‘Islands’, meaning the Channel Islands. The Isle of Man is not part of this grouping, though the judgment in *Barclay* does note that there are similarities in the relationship between the Isle of Man and the UK.

17 There are added complexities that we later touch on that mean this would not necessarily be exactly the same for the Isle of Man, but it broadly holds true.

18 However, there have been points of contention historically of different but related sorts. For example, a 1998 review of financial regulation in the Crown Dependencies by the Home Office. See Philip Morris, ‘Constitutional Practices and British Crown Dependencies: The Gap Between Theory and Practice’ (2012) 41 Common Law World Review 1. Or the far more recent consideration by the UK Parliament around the imposition of public registers of beneficial ownership for the Crown Dependencies (and, indeed, other Overseas Territories). See Peter Clegg, ‘The Crown Dependencies and Overseas Territories: a comparative constitutional and political analysis’ (2025) 63 Commonwealth & Comparative Politics 127.

19 Philip Morris, ‘Constitutional Practices and British Crown Dependencies Revisited: A Case-Study in Implementing Reform by Administrative Guidance’ (2018) 27 Nottingham Law Journal 64. This is, however, the theoretical nature of the relationship. As Morris has argued elsewhere, the traditional framing of the exceptional level of independence enjoyed by the Crown Dependencies per the Kilbrandon Report—see Royal Commission on the Constitution (n 15)—can be considered ‘rather misleading’ when considering certain historic instances. Morris, ibid.

20 s 1(1) British Nationality Act 1981. In this Act, references to the UK are taken to include the Crown Dependencies. s 50(1) British Nationality Act 1981. Further, this arrangement predated the 1981 Act. See Royal Commission on the Constitution (n 15), at para 1347.

21 Cabinet Office and Home Office. 2022. *Common Travel Area guidance*, <https://www.gov.uk/government/publications/common-travel-area-guidance/common-travel-area-guidance> accessed 25 May 2026.

22 Royal Commission on the Constitution (n 15), at para 9.

23 This role was previously carried out by the Home Office.

24 For details of this role, see House of Commons Justice Committee. 2024. *The constitutional relationship with the Crown Dependencies* (HC30).

25 ibid, at para 62.

26 eg ensuring clarity as to an individual’s legal right (or not) to abortion. See *ABC v Ireland* App no 25579/05 (ECtHR 16 December 2010).

27 Royal Commission on the Constitution (n 15), at para 1361.

28 Emily Jackson, *Regulating Reproduction: Law, Technology and Autonomy* (Hart 2001), at 71.

29 Caitlin E Borgmann, ‘Abortion Exceptionalism and Undue Burden Preemption’ (2014) 71Washington and Lee Law Review 1047; Jordan A Parsons and Elizabeth Chloe Romanis, *Early Medical Abortion, Equality of Access, and the Telemedical Imperative* (OUP 2021).

30 The overview we provide here is necessarily brief. For a more detailed exploration, see Parsons and Romanis (n 29); Sally Sheldon and others, *The Abortion Act 1967: A Biography of a UK Law* (CUP 2022).

31 This threshold being considered met at 24 weeks’ gestation. s 37 Human Fertilisation and Embryology Act 1990.

32 [1939] 1 KB 687.

33 s 1(1) Abortion Act 1967.

34 s 1(1)(c) Abortion Act 1967.

35 s 1(1)(b) Abortion Act 1967.

36 s 1(1)(a) Abortion Act 1967.

37 s 1(1)(a) Abortion Act 1967.

38 s 1(1)(d) Abortion Act 1967.

39 Office for Health Improvement and Disparities. 2025. *Abortion statistics, England and Wales: 2022*, <https://www.gov.uk/government/statistics/abortion-statistics-for-england-and-wales-2022/abortion-statistics-england-and-wales-2022>.

40 s 4(2) Abortion Act 1967.

41 [2014] UKSC 68, at paras 37–38.

42 Jordan A Parsons, ‘2017-19 Governmental Decisions to Allow Home Use of Misoprostol for Early Medical Abortion in the UK’ (2020) 124 Health Policy 679.

43 Jordan A Parsons and Elizabeth Chloe Romanis, ‘2020 Developments in the Provision of Early Medical Abortion by Telemedicine in the UK’ (2021) 125 Health Policy 17.

44 s 178 Health and Care Act 2022.

45 s 241 Crime and Policing Act 2026. However, those assisting remain subject to the same legal framework of criminalisation under the Offences Against the Person Act 1861 and Infant Life (Preservation) Act 1929. See also Emily Ottley, ‘New Clause 1 and the Politics of Abortion Law Reform’ (2026) 26 Medical Law International 73.

46 Jayne Kavanagh and Hayley Webb, ‘Parliament Takes a Historic Step toward Decriminalising Abortion—But the Work isn’t Over’ (2025) 389 British Medical Journal r1348.

47 Royal College of Obstetricians and Gynaecologists, Faculty of Sexual and Reproductive Healthcare of the Royal College of Obstetricians and Gynaecologists, Faculty of Public Health and British Society of Abortion Care Providers. 2024. *Involvement of the Police and External Agencies following Abortion, Pregnancy Loss and Unexpected Delivery: Guidance for healthcare staff*, <https://www.rcog.org.uk/media/s3rf2brq/liaison-with-police-guideline-for-nhs-staff-in-womens-health-2.pdf> accessed 25 May 2026.

48 s 254 Crime and Policing Act 2026.

49 OAPA 1861 does not extend to Scotland. s 78 Offences Against the Person Act 1861. However, abortion was—and remains—a criminal offence at common law in similar terms in Scotland. See sch 5, part II, s J1 Explanatory Notes to the Scotland Act 1998.

50 s 7 Abortion Act 1967.

51 Parsons (n 42).

52 Parsons and Romanis (n 43).

53 Scottish Government. 2025. *Review of Abortion Law in Scotland Expert Group: report*, <https://www.gov.scot/publications/review-abortion-law-scotland-expert-group-report/> accessed 25 May 2026.

54 Office for Health Improvement and Disparities (n 39).

55 s 7(3) Abortion Act 1967.

56 See Parsons and Romanis (n 29) 21–22.

57 s 9(2) Northern Ireland (Executive Formation etc) Act 2019. For a discussion, see, Sally Sheldon, Jane O’Neill, Clare Parker and Gayle Davis, ‘‘Too much, too indigestible, too fast’? The decades of struggle for abortion law reform in Northern Ireland’ (2020) 83 Modern Law Review 761.

58 s 25 Criminal Justice (Northern Ireland) Act 1945, as amended by reg 13 The Abortion (Northern Ireland) (No 2) Regulations 2020.

59 Reg 8 The Abortion (Northern Ireland) (No 2) Regulations 2020.

60 s 71(1) The Criminal Code 1872 [as enacted].

61 s 71(2) The Criminal Code 1872 [as enacted].

62 s 72 The Criminal Code 1872 [as enacted].

63 s 58 Offences Against the Person Act 1861.

64 s 71(1) The Criminal Code 1872 [as enacted].

65 s 71(2) The Criminal Code 1872 [as enacted].

66 Reg 11 Abortion (Northern Ireland) (No 2) Regulations 2020.

67 By an amendment to the Crime and Policing Bill introduced by Tonia Antoniazzi MP, which has now become law. This is discussed further later in this article.

68 Scottish Government (n 53).

69 s 3(1) Infanticide and Infant Life (Preservation) Act 1938.

70 s 3(2) Infanticide and Infant Life (Preservation) Act 1938.

71 s 3(1) Infanticide and Infant Life (Preservation) Act 1938.

72 House of Keys Official Report, 24 January 2017, vol 134, no 8, at 1392-92, per Dr Alex Allinson MHK.

73 s 1 Termination of Pregnancy (Medical Defences) Act 1995.

74 ibid.

75 s 2(2) Termination of Pregnancy (Medical Defences) Act 1995.

76 s 3 Termination of Pregnancy (Medical Defences) Act 1995.

77 s 3(1)(a) Termination of Pregnancy (Medical Defences) Act 1995.

78 s 3(2) Termination of Pregnancy (Medical Defences) Act 1995.

79 s 2(1)(a) Termination of Pregnancy (Medical Defences) Act 1995.

80 s 2(1)(b) Termination of Pregnancy (Medical Defences) Act 1995.

81 s 4(1) Termination of Pregnancy (Medical Defences) Act 1995.

82 s 5(2) Termination of Pregnancy (Medical Defences) Act 1995.

83 ibid.

84 s 2(1)(b) Termination of Pregnancy (Medical Defences) Act 1995.

85 House of Keys Official Report, 24 January 2017, vol 134, no 8, at 1307-10.

86 Allinson was granted leave to introduce a bill in January 2017. House of Keys Official Report, 24 January 2017, vol 134, no 8, at 2580.

87 House of Keys Official Report, 13 February 2018, vol 135, no 11, at 2602-04, per Mr Bill Malarkey MHK.

88 Unless, of course, they are an independent. But independent MPs are ordinarily very few with an unusually high number at present owing to Labour Party disciplinary matters.

89 s 29(a) Abortion Reform Act 2019.

90 s 29(c) Abortion Reform Act 2019.

91 Thus, necessitating the repeal of supplementary legislation, such as the ToPMD 1995. s 29(c) Abortion Reform Act 2019.

92 ss 11(2) and 14(1) Abortion Reform Act 2019.

93 s 6(2) Abortion Reform Act 2019.

94 s 11(1) Abortion Reform Act 2019.

95 s 6(4) Abortion Reform Act 2019.

96 s 6(5) Abortion Reform Act 2019.

97 s 6(6) Abortion Reform Act 2019.

98 s 6(7) Abortion Reform Act 2019.

99 It is feasible there will never be case law to test this. Given the size of the jurisdiction, many laws are never tested in court.

100 s 3 Abortion Reform Act 2019.

101 s 6(8)(a) Abortion Reform Act 2019.

102 s 6(8)(b) Abortion Reform Act 2019.

103 s 6(8)(c) Abortion Reform Act 2019.

104 s 6(8)(d)(i) Abortion Reform Act 2019.

105 s 6(8)(d)(ii) Abortion Reform Act 2019.

106 s 13 Abortion Reform Act 2019. During debates, it was acknowledged that questions have been raised as to the legality or otherwise of sex-selective abortion under the AA 1967. House of Keys Official Report, 13 February 2018, vol 135, no 11, at 1410-12, per Alex Allinson MHK. The ARA 2019, then, provides greater clarity on this.

107 s 6(2) Abortion Reform Act 2019.

108 This is explicitly noted in the ARA 2019. s 9 Abortion Reform Act 2019.

109 s 8 Abortion Reform Act 2019.

110 s 8(4) Abortion Reform Act 2019.

111 s 8(5) Abortion Reform Act 2019.

112 s 8(6) Abortion Reform Act 2019. During debates, there was a question of needing to make this a criminal offence ‘rather than just a professional misdemeaner’. House of Keys Official Report, 13 February 2018, vol 135, no 11, at 1455-57, per Cregeen MHK.

113 s 4(2) Abortion Act 1967; reg.12(3) Abortion (Northern Ireland) Regulations 2020.

114 s 6(3) Abortion Reform Act 2019. Indeed, the ARA 2019 went so far as to broaden the scope of healthcare professionals who could perform this role, including nurses and midwives. s 7(1) Abortion Reform Act 2019.

115 House of Keys Official Report, 13 February 2018, vol 135, no 11, at 1592-93, per Lord Brennan.

116 s 6(9) Abortion Reform Act 2019.

117 s 6(10) Abortion Reform Act 2019.

118 s 6(14)(a) Abortion Reform Act 2019.

119 s 219(1) German Criminal Code.

120 ss 19-20 Abortion Reform Act 2019.

121 s 21 Abortion Reform Act 2019.

122 Abortion Services (Safe Access Zones) Act (Northern Ireland) 2023.

123 Public Order Act 2023.

124 Abortion Services (Safe Access Zones) (Scotland) Act 2024.

125 s 23(1) Abortion Reform Act 2019.

126 s 24(1) Abortion Reform Act 2019.

127 ss 23(2) and 24(4) Abortion Reform Act 2019. A £10,000 fine is a level 5 fine on the Isle of Man, which is the highest level.

128 s 23(4)(b) Abortion Reform Act 2019.

129 Catholic News Agency. 2011. *Abby Johnson reveals details of pro-life turnaround and Catholic conversion*, <https://www.catholicnewsagency.com/news/21730/abby-johnson-reveals-details-of-pro-life-turnaround-and-catholic-conversion> accessed 25 May 2026.

130 House of Keys Official Report, 17 December 2024, vol 142, no 7, at 1285.

131 Darbyshire A, for Isle of Man Today. 2025. *‘Safe access zones’ would be a worrying intrusion on human rights says ex-lecturer*, <https://www.iomtoday.co.im/news/safe-access-zones-would-be-a-worrying-intrusion-on-human-rights-says-ex-lecturer-768767> accessed 25 May 2026.

132 Patricia Lohr, Jonathan Lord and Sam Rowlands, ‘How Would Decriminalisation Affect Women’s Health?’ in Sally Sheldon and Kaye Wellings (eds), *Decriminalising Abortion in the UK: What would it mean?* (Policy Press 2020). See also Parsons and Romanis (n 29).

133 Elizabeth Chloe Romanis and Jordan A Parsons, ‘Early Telemedical Abortion, Safeguarding, and Under 18s: A Qualitative Study with Care Providers in England and Wales’ (2023) 49 BMJ Sexual & Reproductive Health 238.

134 Greffier du Roi. 1910. *Order en Conseil raifiant la Loi sur L’Avortement*, <https://guernseylegalresources.gg/CHttpHandler.ashx?documentid=60341>.

135 The Law on Abortion, titled in French in keeping with custom at the time.

136 Greffier du Roi (n 134).

137 Philip Jeune, ‘Guernsey Approves Abortion’ (1996) 348 Lancet 1304.

138 ibid.

139 Then States Member Niall McCathie commented that the change would mean no longer ‘forc[ing] women to go go the mainland to solve their problems’. BBC News. 1996. *Guernsey votes to legalise abortion*, <http://news.bbc.co.uk/onthisday/hi/dates/stories/june/13/newsid_2512000/2512005.stm> accessed 25 May 2026.

140 s 1(a)(i) Abortion (Guernsey) Law 1997 [as enacted].

141 s 1(a)(ii) Abortion (Guernsey) Law 1997 [as enacted].

142 s 1(b) Abortion (Guernsey) Law 1997.

143 s 1 Abortion (Guernsey) Law 1997 [as enacted].

144 s 71 The Criminal Code 1872 [as enacted]. See also section 3.2.

145 s 2 Abortion (Guernsey) Law 1997.

146 s 3(1) Abortion (Guernsey) Law 1997 [as enacted].

147 s 3(1)(a) Abortion (Guernsey) Law 1997.

148 s 3(1)(b) Abortion (Guernsey) Law 1997.

149 s 3(1)(c) Abortion (Guernsey) Law 1997 [as enacted].

150 s 3(1)(d) Abortion (Guernsey) Law 1997 [as enacted].

151 The tail end of the data used for this figure will have fallen within the new, decriminalised provision, but we do not anticipate that this would make a significant difference. Indeed, the 93% figure is in line with the statistical trend dating back to 2006, whereby terminations on the social ground have fluctuated between 92% and 97%. States of Guernsey Public Health Services. 2023. *Guernsey Abortion Statistics 2020-22*, <https://www.gov.gg/CHttpHandler.ashx?id=171605&p=0> accessed 25 May 2026, at p 9.

152 s5 Termination of Pregnancy (Medical Defences) Act 1995.

153 States of Deliberation of the Island of Guernsey. 2021. *The Abortion (Guernsey) (Amendment) Law, 2021 Explanatory Memorandum*, <https://www.gov.gg/CHttpHandler.ashx?id=140077&p=0#:∼:text=THE%20ABORTION%20(GUERNSEY)%20(AMENDMENT)%20LAW%2C%202021&text=EXPLANATORY%20MEMORANDUM%20This%20Law%20amends,woman%20procuring%20her%20own%20miscarriage> accessed 25 May 2026, at p 1.

154 States of Deliberation of the Island of Guernsey Committee for Health & Social Care. 2020a. *Modernisation of the Abortion (Guernsey) Law, 1997*, <https://www.gov.gg/CHttpHandler.ashx?id=123692&p=0> accessed 25 May 2026, at para 1.2.

155 ibid, at para 5.11.

156 States of Deliberation of the Island of Guernsey Committee for Health & Social Care. 2020b. *Modernisation of the Abortion (Guernsey) Law, 1997. Summary of Public Feedback—June 2020*, <https://www.gov.gg/CHttpHandler.ashx?id=126580&p=0> accessed 25 May 2026.

157 States of Deliberation of the Island of Guernsey Committee for Health & Social Care (n 154) at para 1.3.

158 s 1 Abortion (Guernsey) Law 1997, as amended by s 2 Abortion (Guernsey) (Amendment) Law 2021.

159 States of Deliberation of the Island of Guernsey Committee for Health & Social Care (n 154) at para 1.9.

160 s 1(b) Abortion (Guernsey) Law 1997.

161 s 2 Abortion (Guernsey) Law 1997.

162 s 3(1) Abortion (Guernsey) Law 1997, as amended by s 4(a)(i) Abortion (Guernsey) (Amendment) Law 2021.

163 States of Deliberation of the Island of Guernsey Committee for Health & Social Care (n 154) at para 1.6.

164 s 8 Abortion (Guernsey) Law 1997, as amended by s 8 Abortion (Guernsey) (Amendment) Law 2021.

165 s 5(3)(b) Abortion (Guernsey) Law 1997, as amended by s 6(b) Abortion (Guernsey) (Amendment) Law 2021.

166 s 5(3A)(a) Abortion (Guernsey) Law 1997, as amended by s 6(c) Abortion (Guernsey) (Amendment) Law 2021.

167 s 5(5) Abortion (Guernsey) Law 1997, as amended by s 6(d) Abortion (Guernsey) (Amendment) Law 2021.

168 States of Deliberation of the Island of Guernsey Committee for Health & Social Care (n 154) at para 5.28.

169 s 3(1)(c) Abortion (Guernsey) Law 1997, as amended by s 4(a)(ii) Abortion (Guernsey) (Amendment) Law 2021.

170 s 3(1)(d) Abortion (Guernsey) Law 1997, as amended by s.4(a)(iii) Abortion (Guernsey) (Amendment) Law 2021.

171 Bailiwick Express News Team, for Bailiwick Express. 2023. *EXPLAINED: How far behind is Jersey’s abortion law?*, <https://www.bailiwickexpress.com/news/explainer-what-jerseys-current-abortion-law/>.

172 Sir Edward Coke in Coke’s 3 Institutions of the Laws of England, 47 (1797).

173 Bailiwick Express News Team (n 171).

174 art 2(2)(a) Termination of Pregnancy (Jersey) Law 1997.

175 art 2(2)(b) Termination of Pregnancy (Jersey) Law 1997.

176 art 2(2)(c) Termination of Pregnancy (Jersey) Law 1997.

177 art 2(2)(a) Termination of Pregnancy (Jersey) Law 1997.

178 arts 2(2)(a)–(b) Termination of Pregnancy (Jersey) Law 1997.

179 art 5 Termination of Pregnancy (Jersey) Law 1997.

180 For some specialist areas, there may be only one consultant on Jersey. Even for the broader category of paediatricians, the number is very limited. Whilst data are not readily available, a 2023 job advert indicated a team of 11.

181 art 1(1) Termination of Pregnancy (Jersey) Law 1997.

182 art 7 Termination of Pregnancy (Jersey) Law 1997.

183 Public Health Jersey. 2025. *Abortions Report 2024*, <https://www.gov.je/Government/Pages/StatesReports.aspx?ReportID=5948> accessed 25 May 2026.

184 States of Jersey. 2026. *Draft Termination of Pregnancy (Jersey) Law 202-*, <https://statesassembly.je/publications/propositions/2026/p-16-2026> accessed 25 May 2026, art.1(2).

185 Government of Jersey. 2024. *Termination of Pregnancy in Jersey: Public Consultation Feedback Report*, <https://www.gov.je/SiteCollectionDocuments/Health%20and%20wellbeing/ToP%20public%20consultation%20feedback%20report.pdf> accessed 25 May 2026.

186 Government of Jersey. 2025. *Termination of pregnancy: proposed law changes*, <https://www.gov.je/SiteCollectionDocuments/Health%20and%20wellbeing/Termination%20of%20pregnancy%20consultation%20document.pdf> accessed 25 May 2026.

187 States of Jersey (n 184).

188 States of Jersey Official Report, 12 March 2026. Whilst the law has now passed, it is the draft version that remains available online. As such, this is the one we reference throughout.

189 States of Jersey (n 184), art 3(1)(a).

190 ibid, at para 25 [Report].

191 The draft law also aligns with the phase 1 consultation feedback, in which 74% of respondents stated those seeking a termination should not have to justify a need. ibid, at paras 27 and 28 [Report].

192 ibid, at para 35 [Report].

193 ibid, at para 38 [Report].

194 ibid, art 3(1)(b).

195 ibid, art 5(3).

196 ibid, art 10.

197 States of Jersey Official Report, 11 March 2026, at 1630, per Deputy A. Howell.

198 States of Jersey (n 184), at para 8 [Report].

199 See, eg ITV News. 2025. *Jersey women forced to travel for abortion face no financial help unless it is ‘medically necessary’*, <https://www.itv.com/news/channel/2025-08-07/jersey-women-without-medical-need-denied-abortion-funding> accessed 25 May 2026.

200 States of Jersey Official Report, 12 March 2026, at 1000, per Deputy M Tadier.

201 States of Jersey Official Report, 11 March 2026, at 1645, per Deputy A Howell; States of Jersey Official Report, 12 March 2026, at 1015, per Deputy ME Miller.

202 States of Jersey Official Report, 11 March 2026, at 1645, per Deputy A Howell.

203 States of Jersey (n 184) arts 14(1)(e) and 14(2)(a).

204 ibid, at para 43 [Report].

205 ibid.

206 States of Jersey Official Report, 11 March 2026, at 1645, per Deputy A. Howell.

207 ibid, art 21.

208 ibid, arts 6–8.

209 s 241 Crime and Policing Act 2026.

210 States of Jersey Official Report, 11 March 2026, at 1700, per L.M.C. Doublet.

211 States of Jersey Official Report, 12 March 2026, at 0930, per The Attorney General.

212 States of Jersey Official Report, 12 March 2026, 1100, per The Bailiff [vote on Article 17].

213 States of Jersey Official Report, 12 March 2026, at 1000, per Deputy M Tadier.

214 ibid, at para 43 [Report].

215 As discussed in relation to the Isle of Man—and equally applicable to Guernsey—the size and geography of Jersey make travel across the island relatively easy.

216 Lohr and others (n 132); Parsons and Romanis (n 29). See also Gretchen Ely and Nadine Murshid, ‘The Association between Intimate Partner Violence and Distance Traveled to Access Abortion in a Nationally Representative Sample of Abortion Patients’ (2021) 36 Journal of Interpersonal Violence NP663; Abigail RA Aiken and others, ‘Barriers to Accessing Abortion Services and Perspectives on Using Mifepristone and Misoprostol at Home in Great Britain’ (2018) 97 Contraception 177.

217 States of Jersey Official Report, 11 March 2026, at 1715, per Deputy L.M.C. Doublet.

218 Only two voted against.

219 Government of Jersey (n 184), art 24. Given publication timescales, we recognise that this may already have happened.

220 It is not uncommon for a particular policy project to have just a single civil servant working on it, possibly only part time, when a similar initiative in the UK would have a large team.

221 Office for Health Improvement and Disparities (n 39). See also Fiona Bloomer and Kellie O’Dowd, ‘Restricted Access to Abortion in the Republic of Ireland and Northern Ireland: Exploring Abortion Tourism and Barriers to Legal Reform’ (2014) 16 Culture, Health & Sexuality 366; Mary Boyle and Jane McEvoy, ‘Putting Abortion in its Social Context: Northern Irish Women’s Experiences of Abortion in England’ (1998) 2 Health 283.

222 Government of Jersey (n 186), at para 12.

223 Tynwald Court Official Report, Written Questions for the 2021–22 Parliamentary Year [Question W-202201-0244], vol 139, no 21, at 4112-13, per Mr Lawrie Hooper MHK.

224 This is purely anecdotal and is based on discussions with policy professionals in the Crown Dependencies. We note that if this is true, it does present further issues given the very strict regulation of abortion reporting in Great Britain. See Jordan A Parsons, ‘Should We Relax Abortion Reporting Requirements in Great Britain?’ (2025) 33 Health Care Analysis 121.

225 Where the victim chooses to take up such an offer of support, that is. See Jordan A Parsons and Elizabeth Chloe Romanis, ‘The Case for Telemedical Early Medical Abortion in England: Dispelling Adult Safeguarding Concerns’ (2022) 30 Health Care Analysis 73; Jordan A Parsons and Elizabeth Chloe Romanis, ‘“All hands on deck”: A Qualitative Study of Safeguarding and the Transition to Telemedical Abortion Care in England and Wales’ (2024) 348 Social Science & Medicine 116835.

226 States of Jersey Official Report, 11 March 2026, at 1645, per Deputy A. Howell.

227 Parsons and Romanis (n 225).

228 Further, the Crime and Policing Bill is still in the House of Lords at the time of writing so decriminalisation has not yet taken place in England and Wales.

229 At the time of the debate in question, (partial) decriminalisation of abortion in Northern Ireland had not yet happened. As such, abortion remained a criminal offence throughout the UK.

230 House of Keys Official Report, 6 March 2018, vol 135, no 13, at 2590-96, per Mr Martyn Quayle MHK.

231 House of Keys Official Report, 13 February 2018, vol 135, no 11, at 2563-66, per Lord Brennan.

232 States of Deliberation of the Island of Guernsey (n 153) at page 1.

233 Assisted Dying Bill 2023. It is noteworthy, however, that Royal Assent has not yet been granted, despite the bill completing its journey through Tynwald in March 2025. This was due to concerns of the UK Government over compliance with the ECHR, providing an example of the constitutional tension that can arise as a result of the good governance relationship. In April 2026, further amendments were introduced to attempt to satisfy the concerns of the UK Government.

234 Assisted Dying (Jersey) Law 202-.

235 The Terminally Ill Adults (End of Life) Bill 2024-26 failed in the House of Lords in April 2026 due to lack of time. For a brief discussion, see Alexandra Mullock and Richard B. Gibson. 2026. *What might be the Future of the Terminally Ill Adults (End of Life) Bill following its demise in the House of Lords?*, <https://www.slsa.ac.uk/post/what-might-be-the-future-of-the-terminally-ill-adults-end-of-life-bill-following-its-demise-in-the> accessed 25 May 2026.

